# Reduced neurosteroid potentiation of GABA_A_ receptors in epilepsy and depolarized hippocampal neurons

**DOI:** 10.1002/acn3.51023

**Published:** 2020-04-03

**Authors:** Suchitra Joshi, William H. Roden, Jaideep Kapur, Laura A. Jansen

**Affiliations:** ^1^ Department of Neurology University of Virginia Charlottesville Virginia; ^2^ Seattle Children’s Research Institute Seattle Washington; ^3^ Department of Neuroscience University of Virginia Charlottesville Virginia; ^4^ UVA Brain Institute University of Virginia Charlottesville Virginia; ^5^ Department of Neurology Washington University School of Medicine St. Louis Washington

## Abstract

**Objective:**

Neurosteroids regulate neuronal excitability by potentiating *γ*‐aminobutyric acid type‐A receptors (GABARs). In animal models of temporal lobe epilepsy, the neurosteroid sensitivity of GABARs is diminished and GABAR subunit composition is altered. We tested whether similar changes occur in patients with epilepsy and if depolarization‐induced increases in neuronal activity can replicate this effect.

**Methods:**

We determined GABAR *α*4 subunit expression in cortical tissue resected from pediatric epilepsy patients. Modulation of human GABARs by allopregnanolone and Ro15‐4513 was measured in *Xenopus* oocytes using whole‐cell patch clamp. To extend the findings obtained using tissue from epilepsy patients, we evaluated GABAR expression and modulation by allopregnanolone and Ro15‐4513 in cultured rat hippocampal neurons exposed to high extracellular potassium (HK) to increase neuronal activity.

**Results:**

Expression of α4 subunits was increased in pediatric cortical epilepsy specimens encompassing multiple pathologies. The potentiation of GABA‐evoked currents by the neurosteroid allopregnanolone was decreased in *Xenopus* oocytes expressing GABARs isolated from epilepsy patients. Furthermore, receptors isolated from epilepsy but not control tissue were sensitive to potentiation by Ro15‐4513, indicating higher expression of *α*_4_ *β*_x_ *γ*_2_ subunit‐containing receptors. Correspondingly, increasing the activity of cultured rat hippocampal neurons reduced allopregnanolone potentiation of miniature inhibitory postsynaptic currents (mIPSCs), increased modulation of tonic GABAR current by Ro15‐4513, upregulated the surface expression of *α*4 and *γ*2 subunits, and increased the colocalization of *α*4 and *γ*2 subunit immunoreactivity.

**Interpretation:**

These findings suggest that seizure activity‐induced upregulation of *α*_4_ *β*_x_ *γ*_2_ subunit‐containing GABARs could affect the anticonvulsant actions of neurosteroids.

## Introduction

Neurosteroids, the metabolites of gonadal and adrenal steroid hormones, potentiate *γ*‐aminobutyric acid type‐A receptors (GABARs),^1^, ^2^, ^3^ dampen neuronal excitability, and regulate seizure susceptibility.^4^, ^5^, ^6^, ^7^, ^8^, ^9^ However, neurosteroid sensitivity of GABARs in the dentate granule cells and cortical pyramidal neurons of animals with epilepsy is diminished.^10^, ^11^, ^12^, ^13^, ^14^, ^15^ Subunits constituting GABARs are altered in epilepsy models such that the *δ* and *α*1 subunit expression is reduced, whereas that of the *α*4 and *γ*2 subunits is increased.^13^, ^16^, ^17^, ^18^ Furthermore, a larger fraction of synaptic GABARs contain *α*4 and *γ*2 subunits.^13^, ^14^ Altered neurosteroid potentiation of GABAR‐mediated inhibition and associated increased expression of *α*4*γ*2 subunit‐containing receptors has not been described in tissue resected from patients with epilepsy.

Epilepsy‐associated changes in GABAR expression could be due to the effect of increased neuronal activity during recurrent spontaneous seizures or a consequence of the initial epileptogenic injury. Furthermore, increased neuronal activity and synchronization during seizures could induce pathological changes or homeostatic alterations. Homeostatic plasticity involves scaling up of inhibitory neurotransmission following increase in neuronal activity and vice a versa.^19^, ^20^, ^21^, ^22^, ^23^ In contrast to the pathological alterations such as cell death and kindling which increase the likelihood of subsequent seizures, homeostatic plasticity would dampen neuronal activity and reduce the likelihood of seizures. It remains unclear whether the epilepsy‐associated changes in GABARs constitute pathological or homeostatic alterations.^14^, ^24^, ^25^

Prior qRT‐PCR and immunohistochemical studies have revealed increased expression of *α*4 subunits coincident with the onset of spontaneous seizures.^18^, ^26^ Spontaneous seizure activity also appears to transiently upregulate *α*4 subunit expression,^27^ which suggests that the plasticity of *α*4 subunits could be a homeostatic response. However, alterations in multiple cellular signaling pathways and variability of spontaneous seizures in epileptic animals confound these in vivo studies.

Cultured neurons provide a controlled system to study the effect of increased neuronal activity on homeostatic plasticity of GABARs. High extracellular potassium (HK) increases neuronal activity in cultures,^23^ and these neurons have larger GABAR‐mediated synaptic currents, similar to those observed in hippocampal neurons of epileptic animals.^14^, ^28^ This enhancement could be a homeostatic response to curb increased neuronal firing as the potentiation of GABAergic inhibition corresponds with the stabilization of firing rate.^23^ However, whether this plasticity involves upregulation of existing receptors or de novo expression of receptors with altered neurosteroid sensitivity and distinct subunit composition is not known.

In this study, we determined whether neurosteroid modulation of GABARs expressed in human epilepsy is diminished and whether there is a corresponding increase in the expression of *α*4*γ*2 subunit‐containing receptors. Furthermore, we used cultured rat hippocampal neurons to directly evaluate the effects of increased activity on pharmacological properties of GABARs and their subunit composition.

## Material and Methods

### Human cortical brain specimens

Children with intractable epilepsy were evaluated using standard protocols and underwent resective surgery at Seattle Children's Hospital. Informed consent was obtained with the approval of the hospital’s Institutional Review Board to use a portion of the resected tissue for research purposes. Immediately after excision, the tissue was frozen in liquid nitrogen and stored at −80°C. The epileptogenic regions selected for analysis were the “most abnormal” areas identified based on EEG, electrocorticography, imaging, and pathology data (Table 2). Age‐matched, frozen, autopsy‐derived, control brain tissue (postmortem interval <15 h, non‐neurologic causes of death) was obtained from the NICHD Brain and Tissue Bank for Developmental Disorders at the University of Maryland, Baltimore, Maryland (Table 1). A single surgical control specimen was obtained from uninvolved tissue that was unavoidably removed during brain tumor surgery.

**Table 1:** Control brain specimens analyzed in western blotting studies.

Control number	Age	Sex	Brain region	Cause of death	PMI (hours)	Notes
1*	8	M	Temporal	Cardiac Arrhythmia	5	
2*	13.3	M	Occipital	Asphyxia	5	
3*	4.8	F	Frontal	Asthma	15	
4*	4	M	Frontal	n/a	n/a	Normal cortex removed during tumor surgery
5	7.7	M	Occipital	Drowning	12	
6	12.9	M	Occipital	Asthma	15	
7	1.7	M	Parietal	Asthma	10	
8	2.8	F	Parietal	Drowning	12	
9	17.5	M	Frontal	MVC	4	
10	20.3	M	Occipital	MVC	3	

PMI, postmortem interval; MVC, motor vehicle collision.

* Specimens also used in electrophysiology studies.

### Membrane isolation, *Xenopus* oocyte preparation, and injection

The membranes from frozen human cortical tissue were isolated as described previously.^29^ The membrane pellet was suspended in 5 mmol/L glycine and stored frozen at –80°C until use. The ovaries from female *Xenopus laevis* were isolated and dissociated to isolate oocytes (stage V–VI) as described previously.^30^ Oocytes were injected with 50–100 nL of membrane and incubated at 18°C for 1–3 days before use. All *Xenopus* procedures were approved by the Seattle Children’s Institutional Animal Care and Use Committee (IACUC). Same membrane preparations were used to determine GABA EC50, and allopregnanolone and Ro15‐4513 modulation of GABARs.

### Oocyte electrophysiology

Injected oocytes were placed in a recording chamber and bathed in oocyte Ringer’s solution containing 82.5 mmol/L NaCl, 2.5 mmol/L KCl, 2.5 mmol/L CaCl_2_, 1 mmol/L MgCl_2_, and 5 mmol/L Hepes (pH 7.4). Two‐electrode voltage‐clamp recordings were made at room temperature using a GeneClamp 500B amplifier, Digidata 1322A data acquisition system, and pCLAMP9 data analysis software (Axon Instruments, Molecular Devices Corporation, Sunnyvale, CA).^30^

The absolute current amplitudes recorded in this technique of receptor “microtransplantation” are highly variable from one oocyte to another, and are not representative of current amplitudes present in the source tissue.^30^, ^31^ However, the normalized responses of these currents to pharmacologic agents are both reflective of that observed in the source tissue and highly reproducible.

### Neuronal cultures

Rat hippocampal neuronal cultures were prepared according to a protocol approved by the University of Virginia Animal Care and Use Committee. Dissociated hippocampal neuron‐glia co‐cultures were prepared from newborn (P0) rat pups.^23^ Neurons plated at a density of 100 000/35‐mm culture dish and grown in vitro for 10–12 days were used for electrophysiology and immunochemistry. Organotypic hippocampal slice cultures prepared from neonatal (P5‐7) rat pups^32^ and grown in vitro for 8 days were used for biotinylation and Western blotting experiments. The cultures were treated with 10 mmol/L KCl (HK treatment) for 48 h, whereas control cultures were incubated in culture medium without HK for 48 h.

### Western blotting (human tissues)

Frozen cortical tissue was homogenized in 5 mmol/L Tris/HCl (pH 7.4) with 0.32 mol/L sucrose and centrifuged at 3000*g* for 5 min at 4°C. The supernatant was then centrifuged at 40,000*g* for 1 h at 4°C. The resultant membrane pellet was resuspended in 5 mmol/L Tris/HCl (pH 7.4). Standard western blotting procedures were performed to determine 4 subunit expression.^33^

### Biotinylation and Western blotting (hippocampal slice cultures)

Biotinylation of surface proteins and western blotting was performed as described before.^34^

### Cultured neuron electrophysiology

Miniature inhibitory postsynaptic GABAR‐mediated currents (mIPSCs) were recorded from cultured hippocampal pyramidal neurons using whole‐cell patch clamp technique.^34^ After baseline recording the neurons were perfused with an external solution containing allopregnanolone (10 nmol/L) or Ro15‐4513 (300 nmol/L). To measure tonic current, the external solution was supplemented with 3 *μ*mol/L GABA and 10 *μ*mol/L NO711, picrotoxin (50 *μ*mol/L) was applied at the end of the recordings. MiniAnalysis software (Synaptosoft) was used to analyze mIPSCs and tonic current.^34^

### Immunocytochemistry

Labeling of GABAR subunits was performed as described before^35^, ^36^ using dissociated hippocampal neurons in culture. Neurons were visualized using a Nikon Eclipse TE200 fluorescent microscope equipped with a mercury lamp using a 60X, 1.4 N.A. lens. Colocalization was determined using Metamorph imaging software.^36^

## Results

### Diminished neurosteroid sensitivity of human epilepsy GABARs

**Figure 1:**
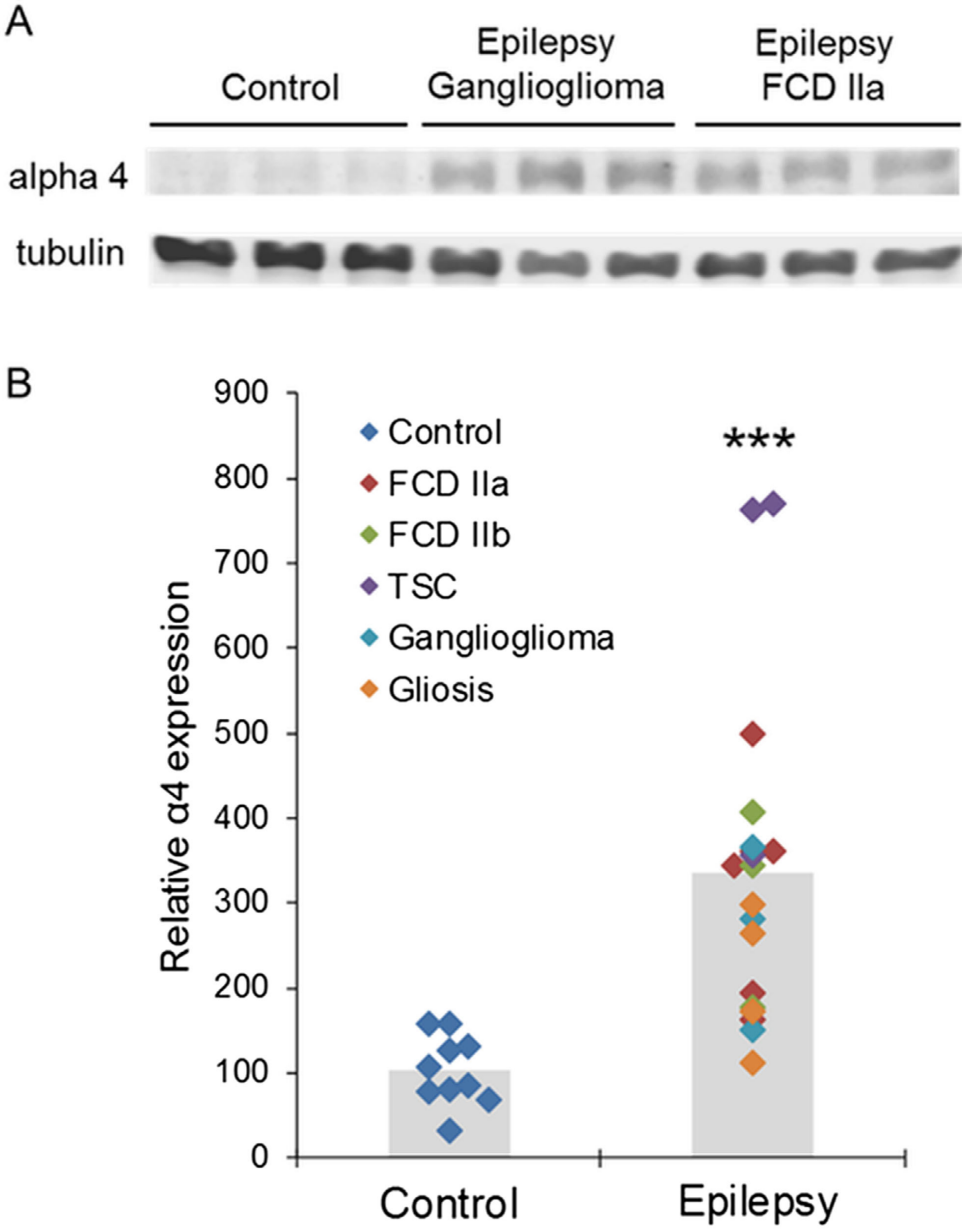
Increased expression of *α*4 subunit‐containing GABARs in human epilepsy. (A) GABAR 4 subunit protein expression in control and epilepsy cortical tissue. A representative blot from three control, three epilepsy‐ganglioglioma, and three epilepsy‐FCD IIa patients is shown. 30 *μ*g of protein was used for each well and *β*‐tubulin expression was used as a loading control. Anti‐*α*4 subunit (1:250 dilution, Novus Biologicals NB300‐194, Littleton, CO) and anti‐*β*‐tubulin (1:2000, Novus Biologicals NB600‐1514) antibodies were used. (B) The expression of *α*4 subunits in epilepsy tissue (336.1 ± 42, *n* = 19) relative to that in control tissue (102.5 ± 12.9, *n* = 10) ***P < 0.001, unpaired *t*‐test.

Cortical brain tissue was collected from control individuals and subjects with epilepsy ranging from 1 to 20 years in age (Control *n* =10, average age 9.3 ± 2.0 years; Epilepsy *n* = 19, average age 8.6 ± 1.0 years, *P* > 0.05, Tables 1 and 2). GABARs in tissue removed from patients with epilepsy are referred to as epilepsy GABARs and those from controls as control GABARs. There was substantially higher *α*4 subunit expression in the tissue isolated from epilepsy patients as compared with control cortex (Fig. 1A). The *α*4 level in epileptic tissue was 330% higher than that in control tissue and was seen across multiple pathologies, including focal cortical dysplasia (FCD) types IIa and IIb, tuberous sclerosis complex (TSC), ganglioglioma, and gliosis (Fig. 1B). This suggests that enhanced *α*4 subunit expression may be due to recurrent seizures rather than the underlying epilepsy etiology.

**Table 2:** Epilepsy brain specimens analyzed in western blotting studies.

Patient number	Age	Sex	Brain Region	Pathology	Clinical Sz. frequency	Seizure types	AED at surgery
1*	7.5	F	Frontal	FCD 2A	2–7/wk	FIAS	ZON, LEV
2*	8	F	Temporal	FCD 2A	4–5/day	FIAS, GTC	LTG, OXC, DIAZ, LRZ
3*	10.8	M	Temporal	FCD 2A	1–4/day	FIAS, GTC	ZON, LTG, CLZ
4*	14.2	F	Temporal	FCD 2A	1/day	FIAS, GTC	LEV, OXC, LTG
5	16	M	Temporal	FCD 2A	1/day	FIAS, FAS	CBZ, LEV
6	5.3	F	Occipital	FCD 2A	10/hour	FIAS	LTG, OXC
7	6.4	F	Frontal	FCD 2B	20/day	FIAS	LEV, TPM
8	8.9	M	Frontal	FCD 2B	2/wk	FIAS	TPM, CLR, ETHO
9	9.4	F	Frontal	FCD 2B	3–10/hour	GTC, FAS	VPA, PHT, MDZ
10	3.3	F	Frontal	TSC	5/day	FIAS, TON	LTG, ZON, LRZ
11	5.1	F	Frontal	TSC	3–4/day	FIAS	CLZ, PHB, TPM
12	18.2	F	Temporal	TSC	1–2/day	FIAS, GTC	OXC, LRZ, GBP
13	4	M	Temporal	GG	unknown	FIAS	LTG
14	5	F	Temporal	GG	4–5/day	FIAS	VPA
15	6.2	M	Temporal	GG	1–2/wk	FIAS	LEV, OXC
16	6.2	M	Frontal	Gliosis	3–5/day	FIAS	LTG, LEV, OXC
17	8.9	M	Temporal	Gliosis	1–2/month	FIAS, GTC	LTG, VPA
18	6.9	F	Temporal	Gliosis	1/day	FIAS	TPM, LTG
19	13.2	M	Temporal	Gliosis	3–4/month	FIAS, GTC	LTG, LEV

AED, antiepileptic drug; CBZ, carbamazepine; CLR, clorazepate; CLZ, clonazepam; DIAZ, diazepam; ETHO, ethotoin; FAS, focal aware seizures; FIAS, focal impaired awareness seizures; FCD, focal cortical dysplasia; GBP, gabapentin; GG, ganglioglioma; GTC, generalized tonic‐clonic seizures; LEV, levetiracetam; LRZ, lorazepam; LTG, lamotrigine; MDZ, midozolam; OXC, oxcarbazepine; PHB, phenobarbital; PHT, phenytoin; TON, tonic seizures; TPM, topiramate; TSC, tuberous sclerosis complex; VPA, valproic acid; ZON, zonisamide.

* Specimens also used in electrophysiology studies.

**Figure 2:**
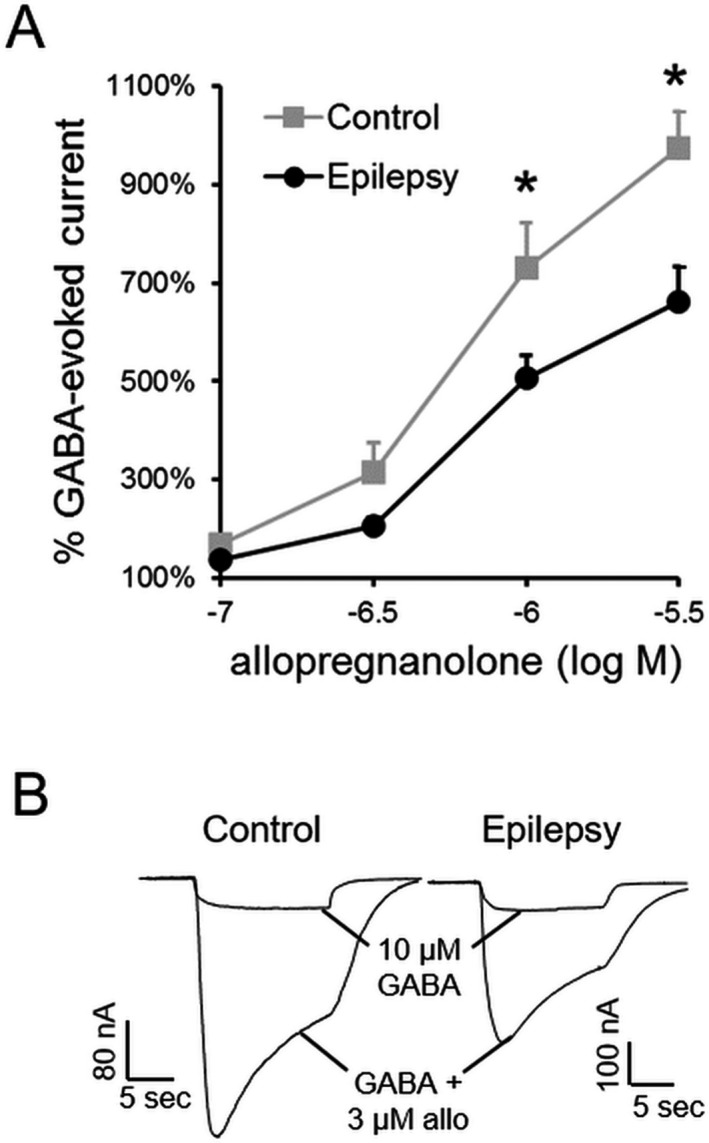
Diminished neurosteroid modulation of GABARs in human epilepsy. (A) Potentiation of GABA‐evoked currents by increasing concentrations of allopregnanolone recorded from oocytes incorporating control and epilepsy GABARs, *n* = 22–26 cells expressing receptors isolated from four control and epilepsy tissues each (*P < 0.05 vs. corresponding control, repeated measures two‐way ANOVA followed by Bonferroni post hoc analysis). At 1 *μ*mol/L allopregnanolone, the potentiation was 730 ± 90% in control tissue and 510 ± 50% in epileptic tissue, and at 3 *μ*mol/L allopregnanolone it was 970 ± 80% versus 660 ± 70%. (B) Representative traces of currents evoked by GABA (10 *μ*mol/L) in the presence or absence of allopregnanolone (3 *μ*mol/L) in an oocyte expressing control or epilepsy GABARs.

We next examined neurosteroid modulation of epilepsy and control GABARs from a subset of the control and epilepsy patients (*n* = 4 each). The epilepsy specimens chosen for analysis all had pathologic diagnoses of FCD IIa to increase sample homogeneity. GABA affinity of control and epilepsy GABARs was similar (EC50 78.4 ± 3.5 *µ*mol/L vs. 63.2 ± 2.6 *µ*mol/L; *n* = 21–25 oocytes expressing receptors isolated from 4 control and epilepsy tissues each). Allopregnanolone potentiation of currents evoked by 10 *µ*mol/L GABA in oocytes incorporating epilepsy GABARs was substantially attenuated compared with control GABARs (Fig. 2A). Thus, epilepsy GABARs were less sensitive to neurosteroids than control GABARs.

**Figure 3:**
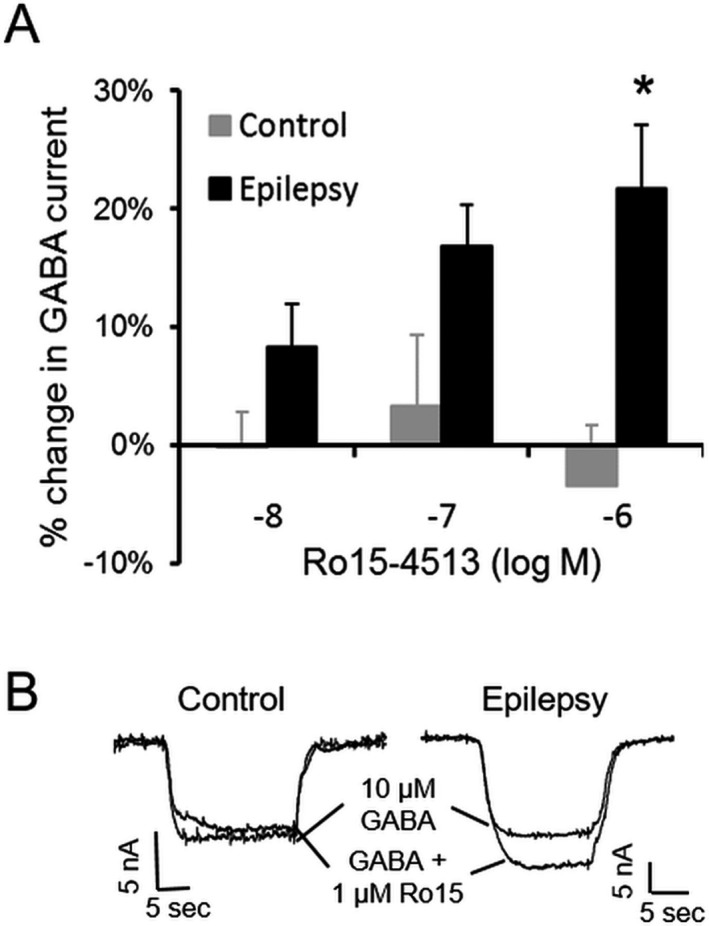
Enhanced modulation of GABAR currents by Ro15‐4513 in human epilepsy. (A) Differential effects of Ro15‐4513 on GABA (10 *μ*mol/L)‐evoked currents in oocytes incorporating control and epilepsy GABARs; *n* = 26–31 cells expressing receptors isolated from four control and epilepsy tissues each. *P < 0.05 versus corresponding control, repeated measures two‐way ANOVA followed by Bonferroni post hoc analysis. (B) Representative traces of currents evoked by GABA (10 *μ*mol/L) in the presence or absence of Ro15‐4513 (1 *μ*mol/L) in an oocyte expressing control or epilepsy GABARs. Ro15‐4513 enhanced the current in oocytes incorporating epilepsy GABARs by 22 ± 5%, whereas it had no effect (−3 ± 5%) on control GABARs.

GABARs containing *α*4 and *γ*2 subunits have unique pharmacological properties, such as being insensitive to benzodiazepines but potentiated by the compound Ro15‐4513. Ro15‐4513 is a partial inverse agonist and inhibits the GABA‐evoked currents of recombinant *α*1*β*x*γ*2 subunit‐containing receptors; however, it is a partial agonist and potentiates the currents of recombinant *α*4*β*x*γ*2 subunit‐containing receptors.^37^, ^38^, ^39^ Because *α*4 subunit expression was increased in epilepsy GABARs, we determined whether this resulted in distinct Ro15‐4513 responses in epilepsy and control GABARs. Application of 10 nmol/L to 1 *μ*mol/L Ro15‐4513 did not affect GABA‐evoked currents recorded from control GABARs, whereas these same concentrations enhanced epilepsy GABAR currents (Fig. 3).

Taken together, these data indicate increased expression of *α*4*γ*2 subunit‐containing GABARs in pediatric epilepsy. However, these studies on human tissue are unable to differentiate GABARs expressed in neuronal versus non‐neuronal cells, and cannot distinguish the effects of seizures from those of underlying pathologies and exposure to antiepileptic medications. Therefore, in subsequent studies, we directly assessed whether increasing neuronal activity in cultured rat hippocampal neurons results in elevated expression of *α*4*γ*2 subunit‐containing GABARs in association with altered responses to neurosteroids and Ro15‐4513.

### Diminished neurosteroid modulation of GABAR‐mediated synaptic currents in High K^+^ (HK)‐treated neurons

Exposure of cultured rat hippocampal neurons to HK causes membrane depolarization and increases neuronal firing.^23^ Perturbations in neuronal activity can induce homeostatic alterations in inhibitory and excitatory neurotransmission, which restore firing rate.^19^ HK‐induced increases in neuronal activity potentiate synaptic GABAR‐mediated neurotransmission.^23^ However, whether this plasticity results from upregulation of existing receptors or from synthesis of different receptor isoforms was not evaluated. We first determined whether GABARs with altered neurosteroid sensitivity were expressed in HK‐treated neurons.

**Figure 4:**
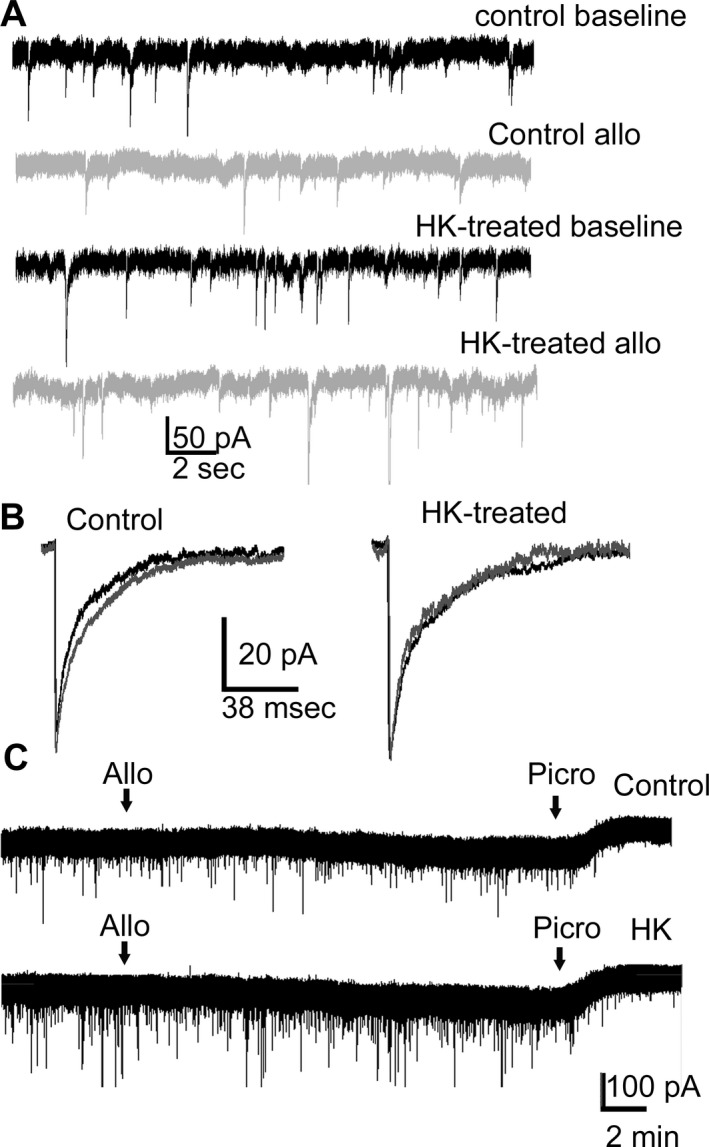
Reduced neurosteroid modulation of synaptic currents in HK‐treated neurons. (A) Representative voltage clamp recordings showing the effect of allopregnanolone (10 nmol/L) in cultured dissociated hippocampal neurons either untreated or treated with HK (10 mmol/L, 48 h). (B) Averaged current from representative control and HK‐treated neurons before (black) and after application of allopregnanolone (gray). Bath application of allopregnanolone (10 nmol/L) prolonged the decay from 35 ± 2 msec to 43 ± 3 msec (*n* = 13, *P* < 0.0005, paired *t*‐test) in control cultures. In contrast, the decay was only prolonged from 29 ± 2 msec to 34 ± 2 msec in the HK‐treated neurons (*n* = 12, *P* < 0.005, paired *t*‐test). All values are presented as mean ± SEM. (C) The allopregnanolone modulation of tonic current in HK‐treated and control neurons. The arrows represent the start of bath application of allopregnanolone (10 nmol/L) and picrotoxin (50 *μ*mol/L). Total tonic current in HK‐treated and control cultures was 25 ± 4 pA, *n* = 5 and 28 ± 3 pA, *n* = 6 neurons, *P* > 0.05. Allopregnanolone enhanced the tonic current by 26 ± 5 pA in HK‐treated neurons (*n* = 14), similar to that in control neurons, 24 ± 3 pA, *n* = 12, *P* > 0.05, *t*‐test.

Allopregnanolone was less effective in potentiating GABAR‐mediated synaptic transmission in HK‐treated neurons compared to control neurons (Fig. 4A and B). Larger amplitude mIPSCs were recorded from the neurons exposed to HK for 2 days indicating homeostatic plasticity; however, allopregnanolone (10 nmol/L) was less effective in prolonging mIPSC decay in these neurons compared untreated controls. Allopregnanolone did not affect the frequency, amplitude, or 10–90% rise‐time of mIPSCs in control or HK‐treated neurons (Table 3), consistent with previous studies, which have found that allopregnanolone at lower doses prolongs the decay and at higher doses increases the amplitude of the current.^14^, ^40^

**Table 3:** Miniature inhibitory postsynaptic currents recorded from HK‐treated (10 mM KCl, 48 h) or untreated neurons by allopregnanolone (10 nmol/L, allo) or Ro15‐4513 (300 nmol/L, Ro).

	Amplitude (pA)		Frequency (Hz)		10–90% rise time (msec)		Weighted decay (msec)	
	Pre‐allo	Post‐allo	Pre‐allo	Post‐allo	Pre‐allo	Post‐allo	Pre‐allo	Post‐allo
Control (*n* = 13)	53 ± 1	55 ± 3	0.26 ± 0.06	0.23 ± 0.06	2.3 ± 0.2	2.3 ± 0.3	35 ± 2	43 ± 3^†^
HK (*n* = 12)	64 ± 4*	63 ± 4	0.42 ± 0.06	0.39 ± 0.07	2.0 ± 0.2	1.6 ± 0.1	29 ± 2*	34 ± 2^†^

	Pre‐Ro	Post‐Ro	Pre‐Ro	Post‐Ro	Pre‐Ro	Post‐Ro	Pre‐Ro	Post‐Ro
Control (*n* = 6)	50 ± 3	53 ± 3	0.23 ± 0.06	0.27 ± 0.07	2.3 ± 0.1	2.2 ± 0.2	42 ± 5	37 ± 5^†^
HK (*n* = 7)	69 ± 6*	66 ± 4	0.31 ± 0.1	0.23 ± 0.06	2.3 ± 0.08	2.3 ± 0.09	40 ± 4	42 ± 6

* *P* < 0.05 versus control (*t*‐test).

^†^ *P* < 0.05 versus predrug (paired *t*‐test).

We then determined allopregnanolone modulation of tonic GABAR currents in HK‐treated neurons. In contrast to potentiation of synaptic currents, HK treatment did not affect allopregnanolone modulation of total tonic current. Allopregnanolone increased the tonic current of control and HK‐treated neurons in a similar manner (Fig. 4C). Thus, the neurosteroid potentiation of tonic GABAR currents was preserved in HK‐treated neurons.

### HK treatment alters GABAR isoforms

**Figure 5:**
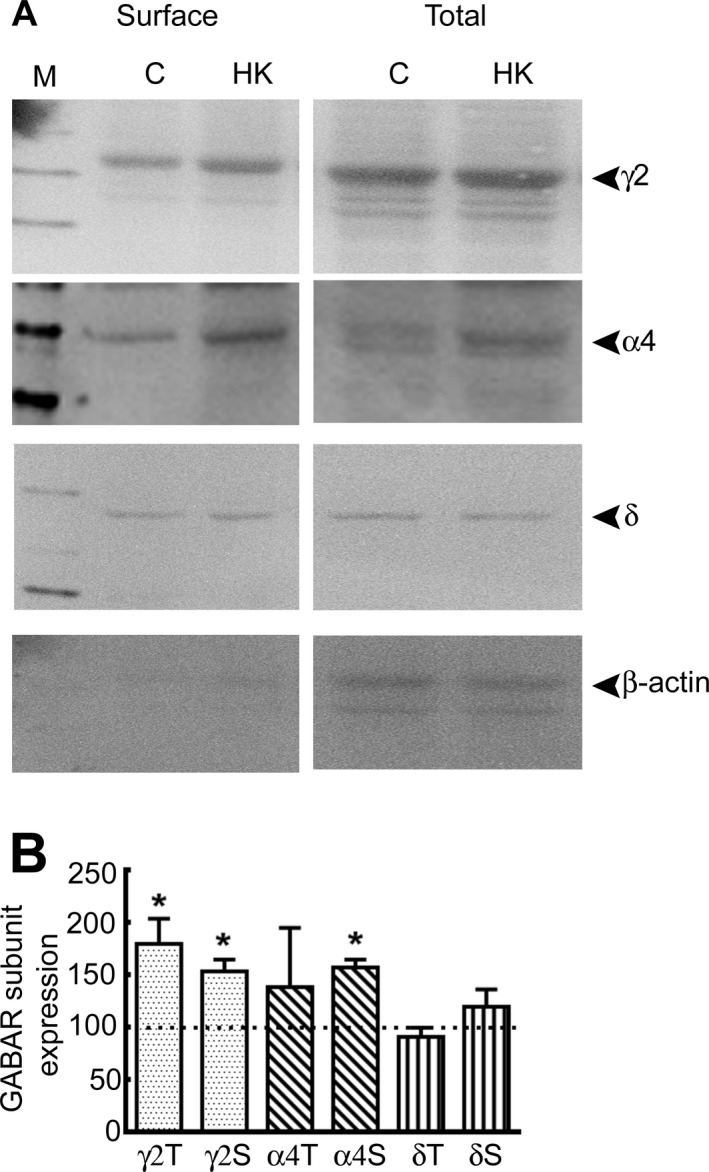
Increased surface expression of *γ*2 and *α*4 subunits in HK‐treated cultures. (A) Representative western blots showing the surface and total expression of the *γ*2, *α*4, and *δ* subunits in HK‐treated (HK) and control (C) organotypic hippocampal slice cultures. The expression of *β*‐actin is shown to demonstrate the purity of the surface samples. Mouse monoclonal anti‐*γ*2 subunit antibody 10F10‐C1‐B8 (2 *μ*g/mL dilution),^49^ rabbit polyclonal anti‐*α4* subunit antibody (1:400 dilution, Millipore, Billerica, MA), anti‐ subunit antibody (1:1000, Millipore, Billerica, MA), and anti‐*β*‐actin (1:5000, clone AC‐40, Sigma‐Aldrich) were used. Lane M shows molecular weight marker. (B) Total (T) and surface (S) expression of *γ*2, *α*4, and *δ* subunits in HK‐treated slice cultures. Data represent average and standard error from *n* = 11 for *γ*2 subunits, *n* = 6 for *δ* subunits and *n* = 4 for *α*4 subunits, *P < 0.05. The subunit protein expression in HK‐treated cultures was normalized to that in control cultures which were run in parallel in every experiment. The total *γ*2 subunit expression in HK‐treated cultures was 179 ± 25% of that in control cultures (*P* < 0.05, Mann–Whitney test), the *δ* subunit expression was 91 ± 10% of that in controls (*P* > 0.05), and that of the *α*4 subunit was 139 ± 56% of that in controls (*P* > 0.05). The normalized surface expression (S) of *γ*2 subunits with HK treatment was 153 ± 11% of that in controls (*P* < 0.05, Mann–Whitney test, Fig. 4B), the *α*4 subunit surface expression in HK‐treated neurons was 158 ± 7%, *P* < 0.05, and the *δ* surface expression of the subunit in HK‐treated neurons was 121 ± 16%, *P* > 0.05.

Altered synaptic GABAR subunit composition can explain the observed diminution in neurosteroid sensitivity of synaptic currents in HK‐treated neurons. The diminished neurosteroid sensitivity of GABAergic synaptic neurotransmission of DGCs from animals with epilepsy is associated with increased expression of *α*4*γ*2 subunit‐containing GABARs.^13^, ^15^, ^18^ Since receptors expressed on the cell surface regulate the pharmacological properties of GABARs, we determined whether the surface expression of GABAR subunits was altered following HK treatment using a biotinylation assay. Higher amounts of *γ*2 and *α*4 subunits were expressed on the neuronal surface membrane in HK‐treated cultures compared to controls. Furthermore, total *γ*2 subunit protein expression was higher in HK‐treated hippocampal slice cultures compared to control cultures, whereas that of the *α*4 subunits was not significantly changed (Fig. 5A). In contrast, surface and total expression of the subunit in HK‐treated and control cultures was similar (Fig. 5A and B).

**Figure 6:**
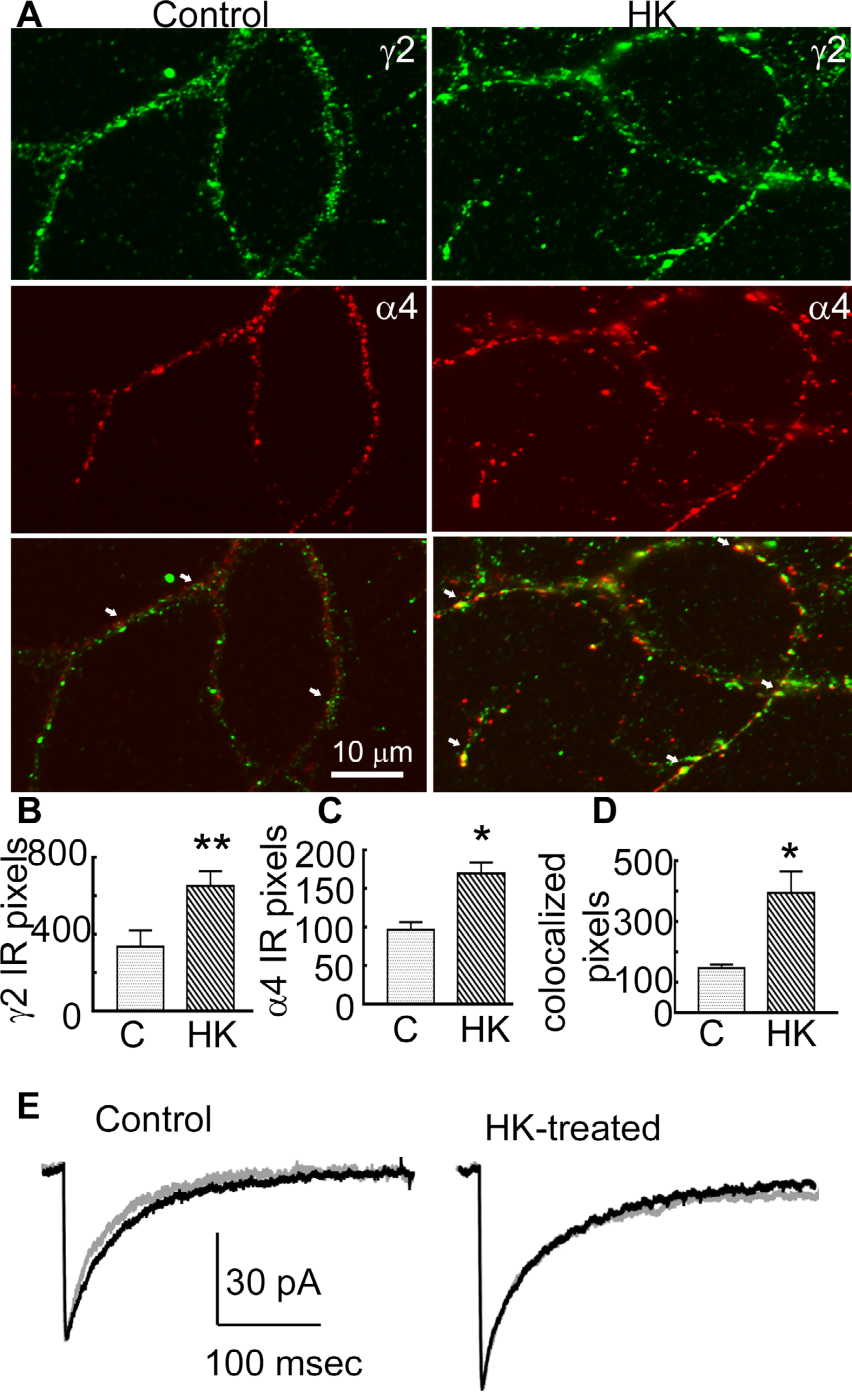
HK treatment increased co‐localization between *γ*2 and *α*4 subunits. (A) Images demonstrating surface immunoreactivity of the *γ*2 (green) and *α*4 (red) subunits and their colocalization over dendrites of representative control and HK‐treated neurons. Arrows mark colocalization of the immunoreactive puncta. (B and C) Number of pixels corresponding to the *γ*2 and *α*4 subunit immunoreactivity in control and HK‐treated neurons, respectively, *n* = 16 control neurons from four batches of cultures and *n* = 26 HK‐treated neurons from 4 batches of cultures, *P < 0.05, **P < 0.005. The average number of *γ*2 puncta per neuron in controls was 338 ± 84 (*n* = 16 neurons from 4 replicate cultures), and in HK‐treated neurons it was 654 ± 76 (*n* = 26 from 4 replicate cultures, *P* < 0.005, *t*‐test). The number of *α*4 subunit puncta was also higher with 170 ± 14 in HK‐treated neurons compared to 97 ± 9 in control neurons (*P* < 0.05). (D) The number of pixels at which the *γ*2 and *α*4 subunit immunoreactivities colocalized, the number of replicates is identical as that in panel B and C, *P < 0.05. The average number of colocalized puncta was 146 ± 13 in control neurons and 395 ± 71 in HK‐treated neurons. (E) Averaged currents illustrating the effect of Ro15‐4513 (300 nmol/L, grey trace) on mIPSCs recorded from a representative control and HK‐treated neuron. The black trace illustrates current before the application of Ro15‐4513. In the control neurons decay shortened from 42 ± 5 msec to 37 ± 5 msec (*n* = 6 neurons/6 replicate cultures, *P* < 0.05, paired *t*‐test), whereas it remained stable in HK‐treated neurons (40 ± 4 msec and 42 ± 6 msec, *n* = 7 neurons/6 replicate cultures, *P* > 0.05, paired *t*‐test). The average change in decay was −5.6 ± 1.5 msec in control neurons and 1.7 ± 1.1 msec in HK‐treated neurons (*P* < 0.05).

Increased surface expression of *α*4 and *γ*2 GABAR subunits suggested potential changes in subunit assembly. To determine whether there was increased synaptic targeting of *α*4*γ*2 subunit‐containing GABARs, we colocalized *α*4 and *γ*2 subunit immunoreactivity on cell membranes of cultured hippocampal neurons. The punctate *γ*2 subunit immunoreactivity was distributed over the soma and dendrites in the control and HK‐treated cultures (Fig. 6A). The *α*4 subunit immunoreactvity was diffusely distributed over cell soma and dendrites of control neurons and only a few clusters were observed (Fig. 6A). In contrast, in HK‐treated cultures the *α*4 subunit immunoreactivity was punctate. In the HK‐treated cultures the *γ*2 and *α*4 subunit immunoreactivity was higher, and the degree of colocalization was also greater than that in controls (Fig. 6A and D).

### Alterations in the effect of Ro15‐4513 in cultured neurons

The immunochemical studies suggested that *α*4*γ*2 subunit‐containing GABARs were assembled in HK‐treated neurons. We directly tested this by determining the effect of Ro15‐4513 on mIPSCs. Ro15‐4513 (300 nmol/L) was bath applied and it shortened the decay of mIPSCs in control neurons, consistent with expression of *α*1*β*x*γ*2 subunit‐containing receptors (Fig. 6E, Table 3). In contrast, Ro15‐4513 did not affect the mIPSC decay in HK‐treated neurons (Fig. 6E, Table 3).

We also tested whether *α*4*β*x*γ*2 subunit‐containing receptors were present at the extrasynaptic membrane. Ro15‐4513 modulation of tonic current was greater in HK‐treated neurons (20 ± 4 pA, *n* = 6, *P* = 0.0004) compared to that in control neurons (5 ± 1 pA, *n* = 10), indicating that some of the *α*4*β*x*γ*2 subunit‐containing receptors were also present at the extrasynaptic sites.

## Discussion

The major findings of this study are (1) GABARs with diminished neurosteroid sensitivity are expressed in cortical tissue isolated from human epilepsy patients, (2) Ro15‐4513 potentiates currents recorded from GABARs expressed in human epilepsy, (3) *α*4 subunit expression is higher in tissue of epilepsy patients than in control tissue, (4) increased neuronal activity triggered by HK treatment upregulates the expression of *α*4*γ*2 subunit‐containing receptors in cultured hippocampal neurons, and (5) HK‐induced neuronal activity diminishes the neurosteroid potentiation of synaptic GABAR‐mediated currents.

Our study demonstrates the expression of GABARs with diminished neurosteroid sensitivity in human epilepsy. This alteration likely renders neurons less protected from seizures. Neurosteroid regulation of GABAR‐mediated inhibition plays a critical role in controlling neuronal activity and keeping seizures under control.^41^ Decreased allopregnanolone levels concomitant with reduced progesterone levels towards the end the menstrual cycle are linked to perimenstrual seizure exacerbation in women with epilepsy.^42^, ^43^ X‐linked epilepsy in PCDH19 patients is also associated with reduced neurosteroid production.^44^ This study performed using tissue from pediatric patients found expression of GABARs with reduced neurosteroid sensitivity. These alterations are likely to impact the anticonvulsant activity of progesterone and could contribute to catamenial seizure exacerbation after menarche in female patients. The pharmacological properties of GABARs expressed in cortical tissue of epilepsy patients were also similar to those composed of α4*γ*2 subunits. These findings complement studies in experimental animals in which increased expression of *α*4*γ*2 subunit‐containing receptors is associated with diminished neurosteroid and diazepam modulation of GABAR currents in DGCs from animals with temporal lobe epilepsy.^12^, ^13^, ^14^, ^15^, ^18^, ^45^, ^46^

These findings are important for treatment considerations. In a recently concluded trial of catamenial epilepsy, progesterone—which exerts anticonvulsant effects via conversion to neurosteroids—was not efficacious in suppressing seizures in women with epilepsy.^47^ The synthetic neurosteroid ganaxolone also has limited therapeutic efficacy for treatment of epilepsy.^48^ The expression of receptors with reduced neurosteroid sensitivity in epilepsy could result in refractoriness to these drugs. However, at the same time, the increased expression of *α*4*γ*2 subunit‐containing GABARs may provide a novel target for treatment. For example, flumazenil, a benzodiazepine‐site ligand with similar pharmacology to Ro15‐4513,^37^ reduces interictal epileptiform activity in patients with intractable epilepsy,^49^ although its concomitant blockade of conventional benzodiazepine‐sensitive GABARs limits its clinical utility.

Several studies have found increased *α*4 subunit expression, accompanied by diminished neurosteroid and diazepam sensitivity, and increased zinc and Ro15‐4513 sensitivity of GABARs in experimental animals with epilepsy.^13^, ^14^, ^15^, ^18^, ^45^, ^46^, ^50^ However, whether the GABARs expressed in human epilepsy have pharmacological properties similar to those reported in experimental animals was unclear. We found markedly increased α4 GABAR subunit expression in tissue isolated from patients with intractable epilepsy and pharmacological alterations similar to those found in epileptic animals. Other human epilepsy tissue studies have also identified either an absolute increase in *α*4 GABAR subunit protein or mRNA expression, or an increase in the *α*4 to *α*1 subunit ratio.^51^, ^52^, ^53^ Our previous work has indicated that other epilepsy‐associated alterations in GABAR subunit expression are pathology‐dependent;^33^ however, the consistent upregulation of *α*4 expression in epilepsy across multiple pathologies suggests that this change may be driven by the increased neuronal activity that occurs during seizures instead of an effect of the underlying etiology of the seizures.

Homeostatic plasticity, strengthening or weakening of synapses, occurs in response to changes in network activity.^19^, ^20^, ^21^, ^22^, ^54^ Increased firing of cultured hippocampal neurons due to depolarization induced by HK potentiates synaptic GABAergic currents.^23^ We found that exposure to HK caused upregulation of *α*4*γ*2 subunit‐containing receptors, likely as a homeostatic response. However, this appears to be an imperfect response, as the pharmacological properties of the receptors were distinct from those expressed in untreated neurons. Low neurosteroid sensitivity of the receptors expressed in HK‐exposed neurons would limit their effectiveness in controlling neuronal excitability.

Ro15‐4513 shortened the decay of mIPSCs recorded from control cultured neurons, whereas it did not affect decay in HK‐treated neurons. These findings agree with the predominant expression of *α*1*γ*2 subunit‐containing receptors in control cultures,^36^ where Ro15‐4513 would be expected to act as a partial inverse agonist, versus a relative increase in *α*4*γ*2 receptors in HK‐treated cultures, where Ro15‐4513 would be expected to act as a partial agonist. The inhibitory effect of Ro15‐4513 on *α*1*γ*2 subunit‐containing receptors was likely counterbalanced by the potentiation of newly expressed *α*4*γ*2 subunit‐containing receptors in HK‐treated neurons. Furthermore, a greater modulation of tonic current of HK‐treated neurons by Ro15‐4513 suggests that some of the *α*4*γ*2 receptors were present at the extrasynaptic membrane. Similarly, Ro15‐4513 had differential actions on GABARs from control versus epilepsy patients, increasing the amplitude of GABA currents mediated by receptors expressed in human epilepsy while having no effect on control receptors. Ro15‐4513 also prolongs the decay of mIPSCs recorded from DGCs of epileptic animals^14^ and causes a greater modulation of tonic current of DGCs of epileptic animals compared to that in control DGCs.^13^

In contrast to synaptic currents, neurosteroid modulation of tonic current was unaffected and the subunit expression was also unchanged in HK‐treated neurons. The *δ* and *α*4 subunits have distinct pharmacological properties.^55^ The neurosteroid modulation of tonic current is mainly dependent on subunit‐containing receptors,^56^ whereas the effect of Ro15‐4513 is dependent on *α*4*γ*2 subunit‐containing receptors. The observed effects of allopregnanolone and Ro15‐4513 on tonic current of HK‐treated neurons are in agreement with preserved subunit and upregulated *α*4*γ*2 subunit expression. Based on these findings, the subunit‐containing GABARs do not appear to contribute to homeostatic plasticity. Studies in experimental animals also suggest that distinct mechanisms regulate the plasticity of *δ*, *α*4, and *γ*2 subunits.^18^, ^26^

HK treatment replicated pharmacological alterations seen in epilepsy patients, but, there appear to be some distinctions in the underlying mechanisms. Total *α*4 subunit expression was increased in the tissue isolated from patients, whereas surface expression but not the total expression of this subunit was upregulated in HK‐treated neurons. It is possible that a longer exposure of cultures to HK conditions may also upregulate the total subunit expression. Exposure to antiepileptic drugs may also have effects on the tissue isolated from patients.

In conclusion, this study revealed the expression of GABARs with diminished neurosteroid sensitivity and altered Ro15‐4513 modulation in both human epileptic cortex and chronically depolarized neurons *in vitro*. These findings indicate that increasing neuronal activity via either recurrent seizures or pharmacologic depolarization triggers plasticity of GABARs, leading to higher expression of receptors containing a combination of *α*4 and *γ*2 subunits. This plasticity may impair the anticonvulsant actions of endogenous and exogenous neurosteroids, but it may also suggest avenues for development of new antiseizure medications.

## Conflict of Interest

The authors do not have any conflict of interest.
